# Association between red blood cell distribution width and 30-day mortality in critically ill septic patients: a propensity score-matched study

**DOI:** 10.1186/s40560-024-00747-x

**Published:** 2024-09-18

**Authors:** Yu-Cheng Wu, Hsin-Hua Chen, Wen-Cheng Chao

**Affiliations:** 1 Department of Critical Care Medicine, Taichung Veterans General Hospital, Taichung City, Taiwan; 2 Doctoral Program in Translational Medicine, National Chung Hsing University, Taichung City, Taiwan; 3 Division of Clinical Informatics, Center of Quality Management, Taichung Veterans General Hospital, Taichung City, Taiwan; 4 Department of post-Baccalaureate Medicine, College of Medicine, National Chung Hsing University, Taichung City, Taiwan; 5 Big Data Center, National Chung Hsing University, Taichung City, Taiwan

**Keywords:** RDW, Sepsis, Biomarker, Mortality, TriNetX

## Abstract

**Background:**

Sepsis is the leading cause of death worldwide, and a number of biomarkers have been developed for early mortality risk stratification. Red blood cell distribution width (RDW) is a routinely available hematological data and has been found to be associated with mortality in a number of diseases; therefore, we aim to address the association between RDW and mortality in critically ill patients with sepsis.

**Methods:**

We analyzed data of critically ill adult patients with sepsis on the TriNetX platform, excluding those with hematologic malignancies, thalassemia, and iron deficiency anemia. Propensity score-matching (PSM) (1:1) was used to mitigate confounding effects, and hazard ratio (HR) with 95% confidence (CI) was calculated to determine the association between RDW and 30-day mortality. We further conducted sensitivity analyses through using distinct cut-points of RDW and severities of sepsis.

**Results:**

A total of 256,387 critically ill septic patients were included in the analysis, and 40.0% of them had RDW equal to or higher than 16%. After PSM, we found that high RDW was associated with an increased 30-day mortality rate (HR: 1.887, 95% CI 1.847–1.928). The associations were consistent using distinct cut-points of RDW, with the strength of association using cut-points of 12%, 14%, 16%, 18% and 20% were 2.098, 2.204, 1.887, 1.809 and 1.932, respectively. Furthermore, we found consistent associations among critically ill septic patients with distinct severities, with the association among those with shock, receiving mechanical ventilation, bacteremia and requirement of hemodialysis being 1.731, 1.735, 2.380 and 1.979, respectively.

**Conclusion:**

We found that RDW was associated with 30-day mortality in critically ill septic patients, underscoring the potential as a prognostic marker in sepsis. More studies are needed to explore the underlying mechanisms.

**Supplementary Information:**

The online version contains supplementary material available at 10.1186/s40560-024-00747-x.

## Introduction

Sepsis presents a global health challenge due to the high mortality rate and is responsible for nearly 20% of global deaths, emphasizing the need for early identification of mortality risk [1, 2]. Several mortality-relevant biomarkers have been identified in patients with sepsis, and these markers represent a promising avenue for improving sepsis management, such as C-reactive protein, procalcitonin, presepsin, protein C, monocyte chemo-attractant protein-1, and angiotensin [3–7]. However, the majority of aforementioned biomarkers were only available in studies for research purposes and cannot be available in the clinical practice in the management of patients with sepsis.

Red blood cell distribution width (RDW) is an easily accessible biomarker obtained from routine hematologic tests and has emerged as a prognostic indicator across various conditions, particularly infectious, inflammatory, and cardiovascular diseases [8–10]. Recent evidence has shown that high RDW was associated with a poor outcome in critically ill patients, including those with acute respiratory distress syndrome, pulmonary embolism, septic shock, influenza, and coronavirus disease 2019 [11–18]. Zhang et al. analyzed data from 11 studies, primarily involving septic patients in Asia, and found that elevated RDW was associated with a slight increase in mortality among sepsis patients [19]. However, the high heterogeneity among these studies, coupled with confounding factors such as hemoglobin levels and blood transfusion practices, limit the robustness of these findings [20–23]. Additionally, inconsistencies in the severity of sepsis and the RDW cut-off points used across studies further make it more challenging to interpret the prognostic value of RDW. Therefore, our study aims to address these gaps by utilizing a large cohort of critically ill septic patients, employing propensity score-matching (PSM), and examining various RDW cut-off points and sepsis severities to elucidate the association between RDW and mortality in this population.

## Methods

### Data sources

We use data and analytic tools on TriNetX, which is a federated real-world data and analytics platform for research [24]. In brief, TriNetX integrated clinical data from various sources, including electronic health records and insurance claims from 118 health organizations (HCOs) in the Global Collaborative Network and supported advanced analytic tools, such as PSM. The TriNetX platform ensured the integration of large-scale real-world data while adhering to patient privacy regulations. Our specific use of TriNetX for this study received approval from the institutional review board committee of Taichung Veterans General Hospital (SE22220A and CE24065C). We queried data and conducted analyses on the TriNetX Research network platform on July 17, 2024. We analyzed a historical data set that includes patient demographics, diagnoses (using International Classification of Disease, Tenth Revision (ICD-10) Clinical Modification coding), medical procedures (coded by ICD-10 Procedure Coding System or Current Procedural Terminology), laboratory tests (Logical Observation Identifiers Names and Codes (LOINC) coded), and healthcare service usage (Supplement Table 1 for the detailed codes).

### Inclusion and exclusion criteria

The inclusion criteria are patients: (1) adults with the ICD code of sepsis between 2010 and 2022; (2) had received critical care service; and (3) had data of RDW. The exclusion criteria are patients who had a history of (1) hematologic malignancy; (2) thalassemia; and (3) iron deficiency anemia. The index date in this study was the diagnosis of sepsis. The targeted exposure was the level of RDW measured within 7 days on or after the sepsis. The time window of laboratory data and hemodynamic data was the same as the time window of RDW, and the presence of comorbidity was defined as a medical visit with the comorbidities within 6 months on or before the diagnosis of sepsis. Given that RDW is a routinely measured clinical parameter and sepsis patients may have multiple RDW records, we excluded those with multiple RDW values, resulting in conflicting classifications at different cut-off points. Subsequently, this cohort was divided based on RDW levels into two balanced subgroups: those with RDW lower than 16% (60.0%) and those with RDW equal to or higher than 16% (40.0%).

### Covariates

To reduce the potential confounding effects, we matched demographics, including age, sex, ethnicity, smoking status as well as a history of alcoholism, and comorbidities consisting of hypertension, diabetes mellitus, heart failure, cerebrovascular disease, asthma, chronic obstructive pulmonary disease, chronic kidney disease, liver fibrosis, presence of neoplasm and metastatic solid tumor. Previous studies have shown that patients with autoimmune disease had high RDW, so we matched the autoimmune diseases, including rheumatoid arthritis, systemic lupus erythematosus, and ankylosing spondylitis, in this study. Moreover, we matched variables in the Acute Physiology and Chronic Health Evaluation (APACHE) II score, including white blood cells, hemoglobin, hematocrit, platelets, albumin, creatinine, sodium, potassium, body temperature, heart rate, pH in serum, blood pressure, respiratory rate and inhaled oxygen concentration. We also matched red blood cell-relevant covariates, including blood transfusion prior to diagnosis of sepsis.

### Outcomes and sensitivity analysis

The primary outcome of this study was all-cause 30-day mortality. To validate the robustness of the association between RDW and mortality in critically ill septic patients, we performed sensitivity analyses using different RDW cut-points (12%, 14%, 16%, 18%, and 20%) and across various severities of sepsis. The severities of sepsis included shock (defined by vasopressor use), receiving mechanical ventilation, the presence of bacteremia, and the need for hemodialysis.

### Statistical analysis

The descriptive results were presented as means ± standard deviation or number (percentages). The risk difference, risk ratio, and crude odds ratio between the high and low RDW groups, using 16% as the cut-point, were calculated. The association between RDW and mortality was illustrated using the Kaplan–Meier plot. The 1:1 PSM was employed through greedy nearest neighbor matching with a caliper width of 0.20 [25]. The hazard ratio (HR) with 95% confidence (CI) was calculated to determine the association between RDW and 30-day mortality in critically ill septic patients.

## Results

### Baseline characteristics of this study before and after the matching

**Fig. 1:**
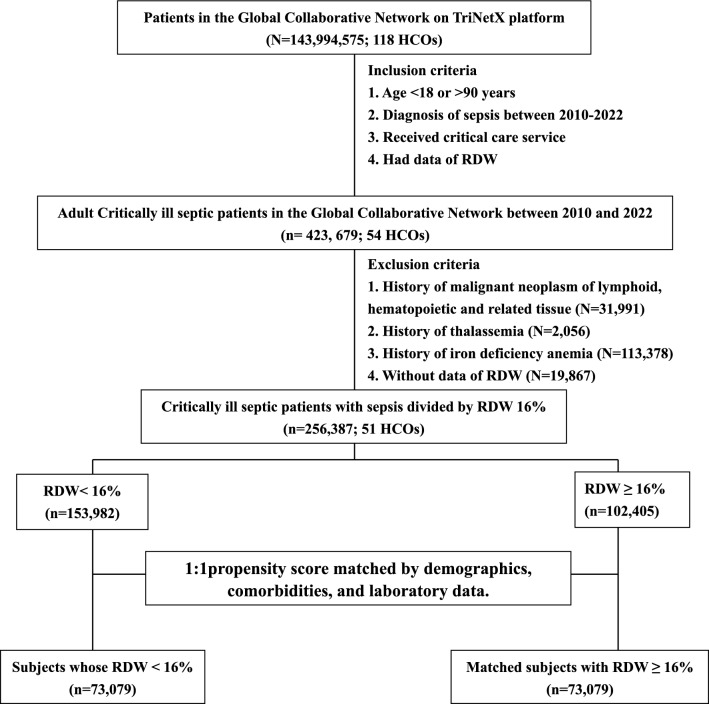
Flow diagram of propensity score-matching. *HCO* healthcare organizations, *RDW* red blood cell distribution width

A total of 256,387 adult critically ill septic patients who were admitted between 2012 and 2022 were included for analyses (Fig. 1). We divided the enrolled patients with sepsis by the RDW 16%, and 40.0% of them were categorized as high RDW. Patients in the high RDW group were more likely to be older, female, African American and had a history of alcoholic consumption, whereas were less likely to be Caucasian, Asian and smoker compared with patients in the low RDW group (Table 1). In the context of comorbidities, patients with high RDW appeared to have comorbidities, except they were less likely to have asthma than those with low RDW. With regard to the laboratory data, patients with low RDW had a higher level of hemoglobin (11.8 ± 2.8 vs 9.7 ± 2.6 g/dL, p < 0.001) and albumin (3.3 ± 0.8 vs 2.8 ± 0.8, *p* < 0.001), lower serum creatinine (1.5 ± 1.7 vs 2.0 ± 2.0 mg/dL, *p* < 0.001), and were less likely to receive blood transfusion prior to sepsis (4.1% vs 10.7%, *p* < 0.001). After 1:1 PSM, 146,158 critically ill septic patients were included to determine the association between RDW and 30-day mortality, and the aforementioned potential confounders were well matched except for slightly high standard mean differences (SMD) in hemoglobin (SMD: 0.159) and albumin (SMD: 0.157).

**Table 1:** Characteristics between patients categorized by red cell distribution in the primary cohort and cohort matched with propensity score-matched cohort

	Before PSM				1:1 PSM			
	RDW≧16%	RDW < 16%	p-value	SMD	RDW≧16%	RDW < 16%	*p*-value	SMD
	*n* = 102,405	*n* = 153,782			*n* = 73,079	*n* = 73,079		
Demographics								
Age, years	68.2 ± 14.3	65.0 ± 16.6	< 0.001	0.210	67.5 ± 14.9	67.6 ± 15.4	0.106	0.008
Sex (male)	54.1%	58.0%	< 0.001	0.080	55.3%	55.1%	0.529	0.003
Ethnicities								
Caucasian	66.1%	71.4%	< 0.001	0.115	68.6%	69.1%	0.033	0.011
African American	19.0%	13.0%	< 0.001	0.164	16.6%	16.3%	0.138	0.008
Asian	2.9%	4.1%	< 0.001	0.064	3.2%	3.2%	0.720	0.002
Other races	2.5%	3.2%	< 0.001	0.042	2.6%	2.7%	0.659	0.002
Smoking	23.4%	24.7%	< 0.001	0.032	23.9%	23.8%	0.618	0.003
Alcoholic	12.5%	11.2%	< 0.001	0.038	11.8%	11.7%	0.541	0.003
Comorbidities								
Essential hypertension	58.2%	57.6%	< 0.001	0.011	58.8%	59.2%	0.067	0.009
Type 2 diabetes mellitus	40.6%	36.2%	< 0.001	0.092	39.4%	39.5%	0.549	0.003
Heart failure	38.6%	25.2%	< 0.001	0.289	34.2%	34.3%	0.624	0.002
Cerebrovascular disease	21.5%	19.0%	< 0.001	0.064	21.0%	21.1%	0.710	0.002
Ischemic heart disease	39.7%	31.7%	< 0.001	0.168	37.5%	37.7%	0.584	0.003
Asthma	8.8%	10.4%	< 0.001	0.052	9.3%	9.2%	0.481	0.004
COPD	24.8%	20.6%	< 0.001	0.099	23.7%	23.9%	0.465	0.004
Chronic kidney disease	34.4%	21.3%	< 0.001	0.295	29.8%	29.8%	0.978	< 0.001
Fibrosis and cirrhosis of liver	11.8%	4.0%	< 0.001	0.294	7.7%	7.2%	< 0.001	0.020
Rheumatoid arthritis	0.6%	0.4%	< 0.001	0.022	0.6%	0.6%	0.764	0.002
Systemic connective tissue disorders	1.3%	0.9%	< 0.001	0.038	1.1%	1.1%	0.981	< 0.001
Ankylosing spondylitis	0.2%	0.2%	0.194	0.004	0.2%	0.2%	0.538	0.003
Neoplasm	33.0%	20.9%	< 0.001	0.275	28.7%	28.3%	0.072	0.009
Metastatic solid tumor	10.0%	3.5%	< 0.001	0.262	6.8%	6.3%	< 0.001	0.021
Glasgow Coma Scale, total score	1.9%	2.1%	< 0.001	0.014				
Laboratory and hemodynamic data								
White blood cell count (10^3^/μL)	13.3 ± 9.3	13.2 ± 10.5	0.036	0.009	13.4 ± 9.3	12.9 ± 8.0	0.623	0.002
Hemoglobin (g/dL)	9.7 ± 2.6	11.8 ± 2.8	< 0.001	0.789	10.2 ± 2.6	10.6 ± 2.8	< 0.001	0.159
Platelet (10^3^/μL)	218.5 ± 141.7	231.9 ± 116.8	< 0.001	0.103	225.8 ± 141.3	229.0 ± 128.5	0.074	0.009
Albumin (mg/dL)	2.8 ± 0.8	3.3 ± 0.8	< 0.001	0.601	2.9 ± 0.8	3.0 ± 0.8	< 0.001	0.157
Creatinine (mg/dL)	2.0 ± 2.0	1.5 ± 1.7	< 0.001	0.058	1.9 ± 2.0	1.7 ± 1.9	0.576	0.003
Sodium (mmol/L)	136.8 ± 6.4	136.4 ± 5.9	< 0.001	0.104	136.9 ± 6.4	136.4 ± 6.0	0.208	0.006
Potassium (mmol/L)	4.1 ± 0.8	4.0 ± 0.7	< 0.001	0.094	4.1 ± 0.8	4.0 ± 0.7	0.278	0.005
Hematocrit (%)	30.5 ± 7.8	36.1 ± 7.9	< 0.001	0.103	31.9 ± 8.0	32.9 ± 7.9	0.201	0.006
Body temperature (℉)	91.4 ± 18.4	92.0 ± 18.1	< 0.001	0.075	91.4 ± 18.5	91.8 ± 18.2	0.723	0.002
Heart rate (beat/min)	85.2 ± 21.3	85.0 ± 20.8	< 0.001	0.093	85.5 ± 21.4	84.3 ± 20.5	0.689	0.002
pH in serum, plasma or Blood	7.3 ± 0.2	7.3 ± 0.2	< 0.001	0.186	7.3 ± 0.2	7.3 ± 0.2	0.129	0.008
Blood pressure, diastolic (mmHg)	57.8 ± 18.2	62.6 ± 18.4	< 0.001	0.054	58.9 ± 18.2	60.5 ± 18.4	0.906	0.001
Blood pressure, systolic (mmHg)	103.8 ± 28.3	110.0 ± 28.8	< 0.001	0.054	105.0 ± 28.4	107.7 ± 29.1	0.869	0.001
Respiratory rate (breath/min)	16.7 ± 5.2	16.8 ± 4.8	< 0.001	0.101	16.8 ± 5.2	16.7 ± 4.9	0.264	0.006
Inhaled oxygen concentration (FiO2%)	24.2 ± 29.5	24.9 ± 29.4	< 0.106	0.051	24.8 ± 29.9	24.4 ± 28.9	0.901	0.001
Procedure								
Blood transfusion	10.7%	4.1%	< 0.001	0.257	7.6%	7.3%	0.006	0.014

Data are shown as mean ± standard deviation and percentages

*PSM* propensity score-matching, *RDW* red blood cell distribution width, *SMD* standard mean difference, *COPD* chronic obstructive pulmonary disease

### Association between RDW and 30-day mortality in critically ill patients with sepsis

**Fig. 2:**
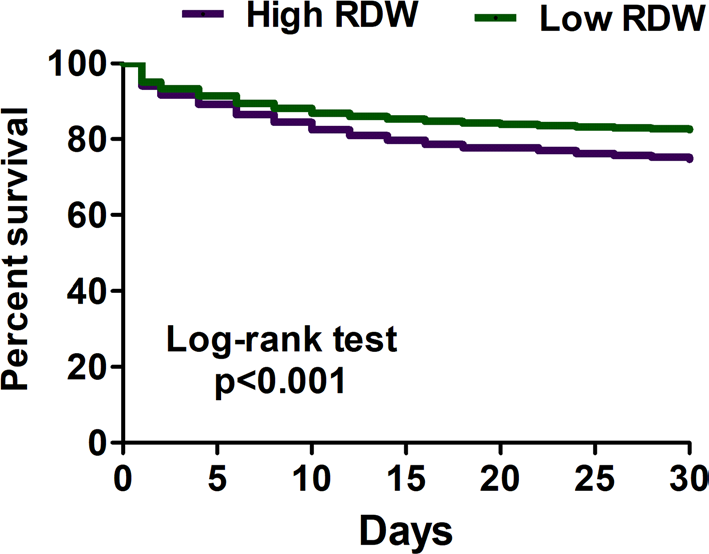
Kaplan–Meier survival curves for 30-day survival among critically ill patients with sepsis stratified by the red cell distribution higher and less than 16%

The risk difference, risk ratio and HR after 1:1 PSM were 0.131 (0.127–0.136), 1.755 (1.722–1.788) and 1.887 (1.847–1.928), respectively (Table 2). We plotted the Kaplan–Meier curve to illustrate the association between RDW and 30-day mortality in critically ill patients with sepsis (Fig. 2). The sensitivity analyses consisted of using different cut-points of RDW and exploring the distinct severities of sepsis. We found that the association between mortality and RDW with distinct cut-points was consistent, and the HR of using RDW 12%, 14%, 16%, 18% and 20% were 2.098 (95% CI 1.717–2.563), 2.204 (95% CI 2.135–2.276), 1.887 (95% CI 1.847–1.928), 1.809 (95% CI 1.769–1.851) and 1.932 (95% CI 1.877–1.989), respectively (Table 3 and Supplemental Fig. 1). We further addressed the association between RDW and distinct severities of sepsis, and the HR of high RDW and 30-day mortality in critically ill septic patients shock, receiving mechanical ventilation, bacteremia and requirement hemodialysis were 1.731 (95% CI 1.672–1.791), 1.735 (95% CI 1.648–1.826), 2.380 (95% CI 2.245–2.523) and 1.979 (95% CI 1.837–2.132), respectively (Table 4).

**Table 2:** Risk of mortality at 30 days in critically ill patients with sepsis classified according to the width of the red cell distribution width

Variable	Total	Event	Risk	Risk difference	Risk ratio	HR ratio
Before propensity score-matching						
RDW < 16%	153,982	21,369	0.139	Reference	Reference	Reference
RDW ≥ 16%	102,405	33,939	0.331	0.193 (0.189, 0.196)	2.388 (2.352, 2.425)	2.655 (2.610, 2.701)
After 1:1 propensity score-matching						
RDW < 16%	73,079	12,987	0.174	Reference	Reference	Reference
RDW ≥ 16%	73,079	22,319	0.305	0.131 (0.127, 0.136)	1.755 (1.722, 1.788)	1.887 (1.847, 1.928)

*PSM* propensity score-matching, *RDW* red blood cell distribution width

**Table 3:** Sensitivity analysis to investigate the association between 30-day mortality and RDW using different cut-off values in the whole cohort and propensity score-matched populations

Cut-off values of RDW	Before 1:1 PSM			After 1:1 PSM		
	Patients with low RDW	Patients with high RDW	HR (95% CI)	Patients with low RDW	Patients with high RDW	HR (95% CI)
12%	2,955	253,083	4.658 (3.950, 5.492)	2,923	2,923	2..098 (1.717, 2.563)
14%	69,597	186,533	3.423 (3.331, 3.518)	61,217	61,217	2.204 (2.135, 2.276)
16%	146,158	102,405	2.655 (2.610, 2.701)	73,079	73,079	1.887 (1.847, 1.928)
18%	213,154	54,897	2.598 (2.554, 2.644)	53,699	53,699	1.809 (1.769, 1.851)
20%	255,397	27,388	2.824 (2.768, 2.882)	27,381	27,381	1.932 (1.877, 1.989)

*PSM* propensity score-matching, *RDW* red blood cell distribution width, *HR* hazard ratio, *CI* confidence interval

**Table 4:** Sensitivity analysis in the estimation of the 30-day mortality risk among critically ill septic patients with distinct severities

Variables	Before PSM			After 1:1 PSM		
	Low RDW (number)	High RDW (number)	HR (95% CI)	High RDW (number)	Low RDW (number)	HR (95% CI)
Sepsis+ICU+shock	29,841	34,458	1.903 (1.851, 1.957)	20,336	20,336	1.731 (1.672, 1.791)
Sepsis+ICU+ ventilator used	18,266	17,770	1.944 (1.866, 2.025)	11,294	11,294	1.735 (1.648, 1.826)
Sepsis +ICU+ bacteremia	28,307	24,196	3.081 (2.939, 3.231)	16,301	16,301	2.380 (2.245, 2.523)
Sepsis+ICU+ hemodialysis	8,439	13,471	2.349 (2.201, 2.506)	7,037	7,037	1.979 (1.837, 2.132)

RDW higher than lower than 16%

*PSM* propensity score-matching, *RDW* red blood cell distribution width, *HR* hazard ratio, *CI* confidence interval

## Discussion

RDW is a ready-to-use hematological parameter in clinical practice among critically ill patients, and increasing evidence have shown the predictive value for adverse outcomes of RDW in a wide range of diseases. In this study, we used a global health research network with a high number of patients and the PSM approach to demonstrate that high RDW was associated with 30-day mortality in critically ill septic patients. The finding appears to be consistent across various severities of sepsis, suggesting the robustness of RDW as a prognostic marker. The relationship between RDW levels and mortality risk further strengthens the utility of RDW in clinical risk stratification, making it a potentially valuable tool for identifying high-risk patients.

The high heterogeneity of sepsis is a substantial issue in the management of patients with sepsis; therefore, there is an essential need to stratify septic patients [26]. Increasing studies have been conducted on clustering septic patients using clinical data and biomarkers, including cytokine profiles and transcriptomic data [27, 28]. Among the numerous biomarkers, those derivable from routine clinical laboratory tests hold particular significance due to the widespread availability and rapid turnaround times, such as C-reactive protein, procalcitonin, and RDW [29]. RDW has been proven to correlate with deleterious outcomes, including mortality, in patients with a wide range of diseases, such as cardiovascular diseases, liver diseases, autoimmune diseases, and malignancies [8, 30–35]. However, sepsis is highly heterogeneous and intersects with many of the aforementioned diseases, necessitating that studies to address the relationship between RDW and sepsis outcomes have to control potential confounding factors meticulously. The present study, for example, excluded patients with a history of hematological diseases and employed PSM to mitigate the confounding effects of comorbidities, thus verifying the relevance of RDW to mortality in critically ill patients with sepsis.

In the research of sepsis, the relationship between RDW and mortality has emerged as a significant area of study, offering insights into prognosis and the potential for early identification of patients at high risk of death. Hunziker et al*.* used the Multiparameter Intelligent Monitoring in Intensive Care II (MIMIC-II) collected from the intensive care units of Beth Israel Deaconess Medical Center from 2001 to 2008 to show that RDW was associated with in-hospital mortality (adjusted OR 1.14, 95% CI 1.08–1.19) [36]. The aforementioned study included a broad cohort of critically ill patients without specifically focusing on sepsis. In contrast, our study used data during 2010–2022 and focused on critically ill patients with sepsis, providing a more targeted analysis of RDW’s prognostic value in this specific population. In line with our study, Kim et al*.* enrolled 329 septic patients who were admitted to the emergency department and identified a positive correlation between RDW and mortality [37]. However, the limited sample size in their study may restrict the generalizability of their findings. Our study addresses this limitation by leveraging the TriNetX platform, which provides access to a large multinational cohort, to explore the association between RDW and mortality in sepsis. Zhang et al. conducted a meta-analysis using data from 17,961 septic patients in 11 studies and reported that high RDW was associated with slightly increased mortality in patients with sepsis (HR 1.14, 95%CI 1.09–1.20) [19]. Zhang et al. acknowledged the high heterogeneity (I^2^ = 80%) in their meta-analysis. Up to 90.9% (10/11) of studies were conducted in Asia, with only one study that cannot be assessed in PubMed, including 11,691 septic patients conducted in the United States [19]. In addition to the heterogeneity, the inability to control potential confounders is an inherent issue in the meta-analysis, given that it is challenging to uniformly control all potential confounders across these studies [38]. In the present study, encompassing 296,475 septic patients, we are able to stringently control RDW-associated confounders by the PSM approach. Furthermore, we focused on critically ill septic patients; therefore, the HR (1.799, 95% CI 1.766–1.832) appears to be higher in this study than those in previous studies among general septic patients without the restriction of critical illness. In this study, we further used distinct severities of sepsis to test the robustness between high RDW and distinct cut-points of RDW to demonstrate the effect of RDW on mortality risk in critically ill patients with sepsis. Collectively, the aforementioned evidence and our data highlight the previously ignored association between high RDW and mortality risk in critically ill septic patients, and RDW, a frequently measured hematological parameter, can be incorporated into early mortality risk stratification in critically ill patients with sepsis.

In critical care research, leveraging large-scale real-world data networks such as TriNetX is crucial for clinically relevant issues that can improve patient classification and may guide clinical practice [39]. However, real-world data often encompass diverse patient demographics, comorbidities, clinical conditions, and treatment modalities. By integrating large-scale real-world data with advanced statistical techniques like PSM, researchers may at least partly mitigate the potential confounding effect and enhance the validity of their findings [40]. As we have shown in this study, the TriNetX database enables us to assess the association between elevated RDW levels and 30-day mortality in critically ill patients with sepsis, and the application of PSM further ensures that the observed relationships are not confounded by other factors.

While not completely understood, several plausible mechanisms, including anemia, oxidative stress, inflammation, vascular damage, and systemic metabolic alterations, have been implicated in the association between high RDW and adverse outcomes [41, 42]. High RDW may indicate anisocytosis associated with anemia, a condition that exacerbates organ dysfunction due to impaired oxygen delivery in septic patients [43]. Oxidative stress, a key feature of sepsis, damages cellular components and increases cell turnover, including red blood cells, making RDW a potential marker for this condition [44]. Additionally, inflammatory mediators contribute to endothelial cell activation, increased vascular permeability, coagulation disruption, and impaired microcirculation, all reflected in high RDW [41]. Metabolic changes induced by sepsis affect nutrient utilization, energy production, and waste elimination, impact red blood cell lifespan and functionality, and lead to RDW variations [42]. For instance, alterations in iron metabolism, a common feature of sepsis, can impact hemoglobin synthesis and red blood cell maturation, leading to changes in RDW, and we hence excluded those with a history of iron deficiency anemia in this study. These evidence highlight the multifactorial nature of sepsis and the potential role of RDW as a marker for the underlying pathophysiological processes. Our findings suggest that RDW may serve as a prognostic tool in sepsis management, warranting further research to elucidate the underlying mechanisms.

There are limitations in this study. First, this study is subject to the inherent limitations of any study reliant on ICD codes; however, one recent study analyzed the accuracy of the ICD coding method to estimate sepsis among 17 studies and reported the sensitivity and specificity were approximately 75% and 85%, respectively [45]. Furthermore, we have used stringent inclusion to minimize the misclassification of patients and performed additional analyses with consistent findings. Second, the level of RDW is not an intervention. Nevertheless, we used the RDW on week one to predict the 30-day mortality, and the application of PSM should be able to mitigate the confounding effect in the observational study. Third, due to the observational design of this study, some unmeasured confounders may exist. Fourth, severity scores, such as APACHE II and Sequential Organ Failure Assessment (SOFA) scores, were unavailable. However, we have matched variables of the APACHE II score and conducted a sensitivity analysis in critically ill septic patients with distinct organ failure, including shock, respiratory failure and renal failure (Table 4). Additionally, some analytic tools, such as Logistical Regression, Inverse Probability of Treatment Weighting (IPTW), covariate-balancing propensity score (CBPS) and restricted cubic spline, are currently unavailable on the TriNetX platform.

## Conclusion

In conclusion, we used data on the TriNetX research network to demonstrate the association between RDW and 30-day mortality in critically ill septic patients, providing evidence for the prognostic significance of RDW in sepsis. These findings indicate the inclusion of RDW in the mortality risk stratification for critically ill patients with sepsis. Further research is warranted to validate our findings and to uncover the mechanistic links between RDW and sepsis outcomes.

## Supplementary Information

Additional file 1.

## Data Availability

The data underlying this article will be shared on reasonable request to the corresponding author.

## Abbreviations

APACHE: Acute Physiology and Chronic Health Evaluation
CBPS: Covariate-balancing propensity score
CI: Confidence interval
COPD: Chronic obstructive pulmonary disease
FiO2: Fraction of inspired oxygen
HCOs: Health organizations
HR: Hazard ratio
ICD: International classification of diseases
ICU: Intensive care unit
LOINC: Logical observation identifiers names and codes
IPTW: Inverse probability of treatment weighting
MIMIC-II: Multiparameter Intelligent Monitoring in Intensive Care II
PSM: Propensity score-matching
RDW: Red blood cell distribution width
SMD: Standard mean difference
SOFA: Sequential Organ Failure Assessment
