# Supplementary material for: Association between red blood cell distribution width and 30-day mortality in critically ill septic patients: a propensity score-matched study
Source: J Intensive Care. 2024 Sep 18;12:34. doi: 10.1186/s40560-024-00747-x (PMC11409593; doi:10.1186/s40560-024-00747-x)
Supplement: Supplementary file 1 — Additional file 1. [file 40560_2024_747_MOESM1_ESM.pdf]

Supplement Table 1. Codes of the variables

Code type	Code	Description
**Including criteria**		
ICD-10	A41.9	Sepsis
CPT	1013729, 1014309, 99291	Critical care
TNX	9008	RDW
**Excluding criteria**		
ICD-10	C81-C96	malignant neoplasms of lymphoid, hematopoietic and related tissue
ICD-10	D56	thalassemia
ICD-10	D50	Iron deficiency anemia
**Characteristics**		
ICD-10	F17	Smoking
ICD-10	F10	alcoholic
ICD-10	I10-I15	Essential hypertension
ICD-10	E11	Type 2 Diabetes mellitus
ICD-10	I50	Heart failure
ICD-10	I60-I69	Cerebrovascular disease
ICD-10	I20-I25	Ischemic heart disease
ICD-10	J45	Asthma
ICD-10	J44	COPD
ICD-10	N18	Chronic kidney disease
ICD-10	K74	Fibrosis and cirrhosis of liver
ICD-10	M05	Rheumatoid arthritis
ICD-10	M32	Systemic lupus erythematosus
ICD-10	M45	Ankylosing spondylitis
ICD-10	C00-D49	Neoplasm
ICD-10	C79	Metastatic solid tumor
ICD-10	R40.24	GCS score, total score
**Lab**		
CPT	9015	White blood cell count (10^3/μl)
CPT	9014	Hemoglobin (g/dL)
CPT	9020	Platelet (10^3/μL)
CPT	9045	Albumin (mg/dL)
CPT	9024	Creatinine (mg/dL)
CPT	9029	Sodium (moles/volume) in serum
CPT	9028	Potassium (moles/volume) in serum
CPT	9013	Hematocrit (volume fraction) of blood
CPT	9076	Body temperature
CPT	9085	Blood pressure, systolic
CPT	9086	Blood pressure, diastolic
CPT	9074	Heart rate

CPT	9073	Respiratory rate
TNX	LG345-5	ABG pH
LOINC	3150-0	Inhaled oxygen concentration (FiO2)
**Procedure**		
CPT	36430	blood transfusion
**Outcome**		
	Deceased	Survival
ICD-10	R57	shock
ICD-10	Z99.11	Dependence on respirator [ventilator] status
ICD-10	R78.81	bacteremia
ICD-10	Z99.2	hemodialysis

Abbreviations: ICD-10, International Classification of Diseases 10th Revision; CPT, Current Procedural Terminology;RDW, Red blood cell distribution width; COPD,Chronic Obstruction Pulmonary Disease.

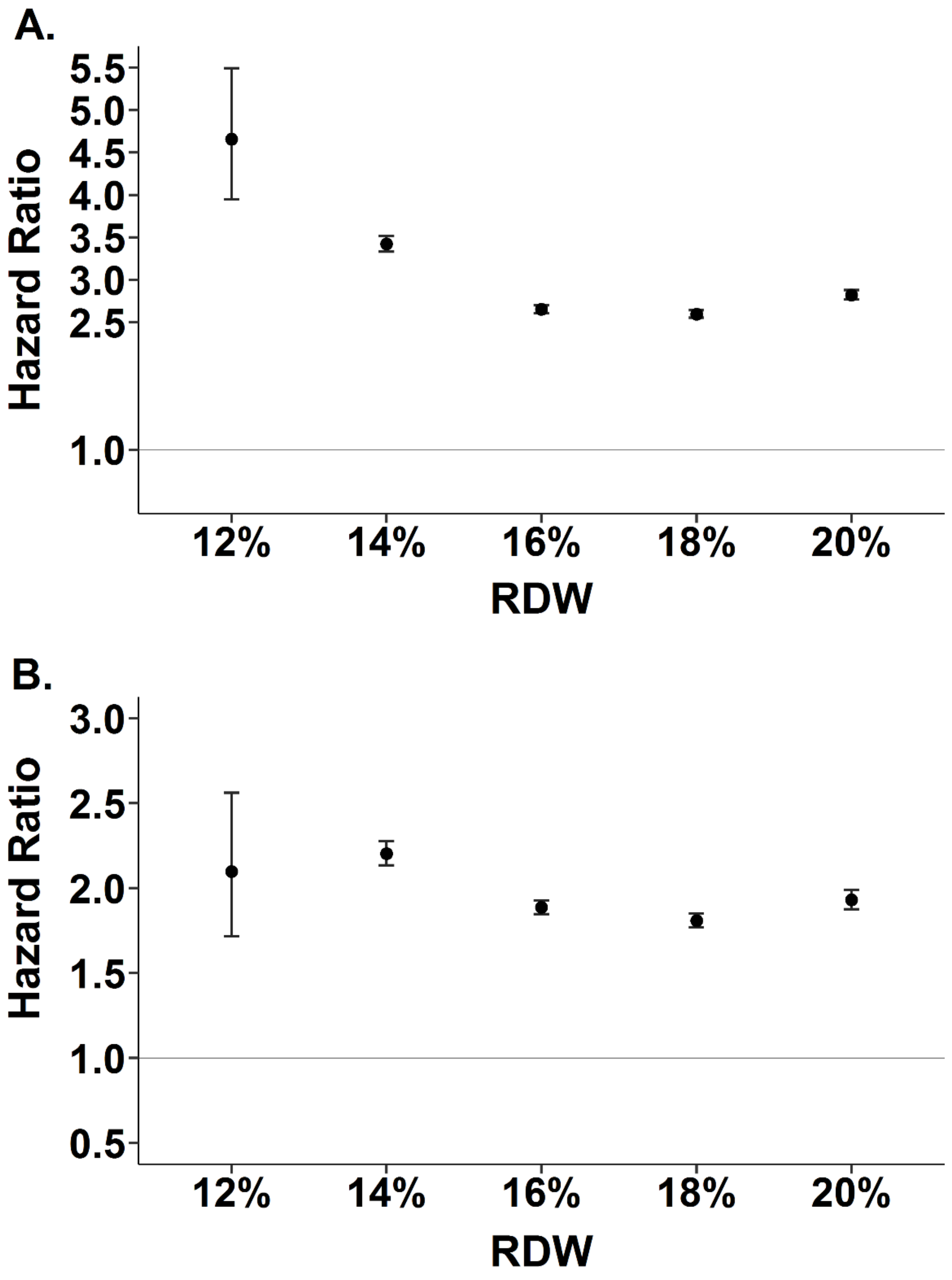

Supplemental Figure 1. Sensitivity analysis to address the association between 30-day mortality and RDW using different cut-off values in the (A) whole cohort and (B) propensity score-matched populations
